# Dermatomyositis Anteceding Lung Adenocarcinoma

**DOI:** 10.7759/cureus.11580

**Published:** 2020-11-19

**Authors:** Jin Lee Lim, Nor Syahida Yusof, Noor Aliza Md Tarekh, Rozanah Abdul Rahman

**Affiliations:** 1 Respiratory Medicine, Hospital Sultanah Aminah, Johor Bahru, MYS; 2 Pathology, Hospital Sultanah Aminah, Johor Bahru, MYS

**Keywords:** dermatomyositis, lung, adenocarcinoma

## Abstract

Dermatomyositis is often presented as paraneoplastic syndrome. The diagnosis of dermatomyositis can prompt clinicians to further investigate the underlying cause, in particular malignancy. This case report illustrates the association of lung adenocarcinoma and dermatomyositis with antecedent presentation of cutaneous and musculoskeletal manifestations, one year prior to the diagnosis of carcinoma.

## Introduction

Dermatomyositis is an inflammatory myopathy with typical cutaneous presentations including heliotrope rash of the periorbital skin, erythematous scaly plaques on dorsal hands along with periungual telangiectasia, and photosensitive poikilodermatous eruptions. Proximal myopathy is generally symmetrical and progresses within a period of weeks to months [1]. It is associated with increased risk of breast, ovary, lung, colorectal, and lymphoma malignancies [2]. The reported malignancy incidence in dermatomyositis varied from less than 7% to over 30% [3]. Dermatomyositis is a form of paraneoplastic syndromes. However, the incidence of its occurrence in lung cancer is approximately 10% [4]. Other predisposing factors associated with development of dermatomyositis are viral infection and drug side effects. We present a case study that highlights dermatomyositis as an antecedent sign of lung adenocarcinoma.

## Case presentation

A 61-year-old gentleman, ex-smoker with no past medical illness, presented with breathlessness and cough for one month. He also reported having skin lesions over his eyebrows and progressive weakness of upper and lower limbs for the past one year. 

**Figure 1:**
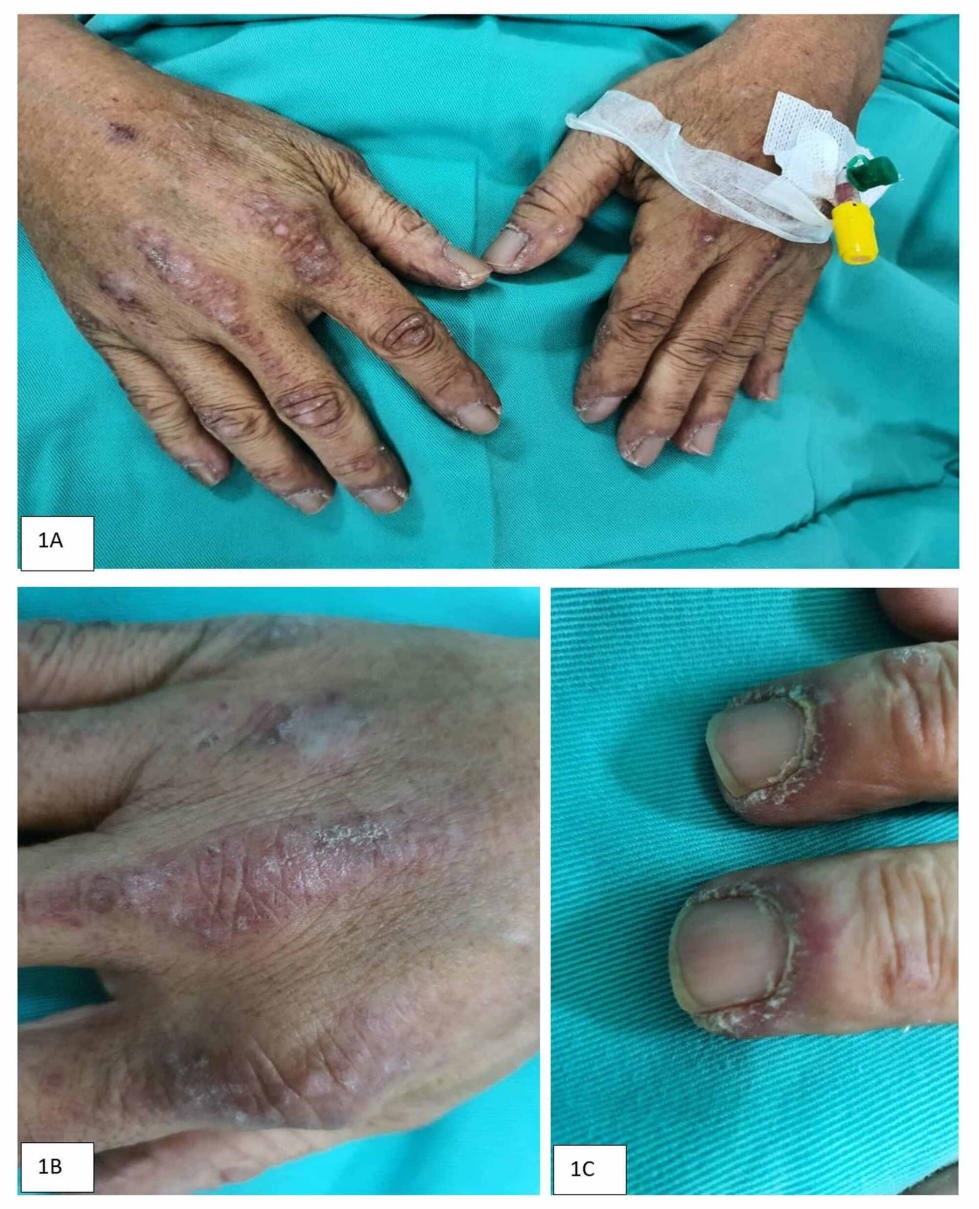
A, B) Gottron’s papules (erythematous popular lesions on the knuckles), C) Periungual telangiectasia

**Figure 2:**
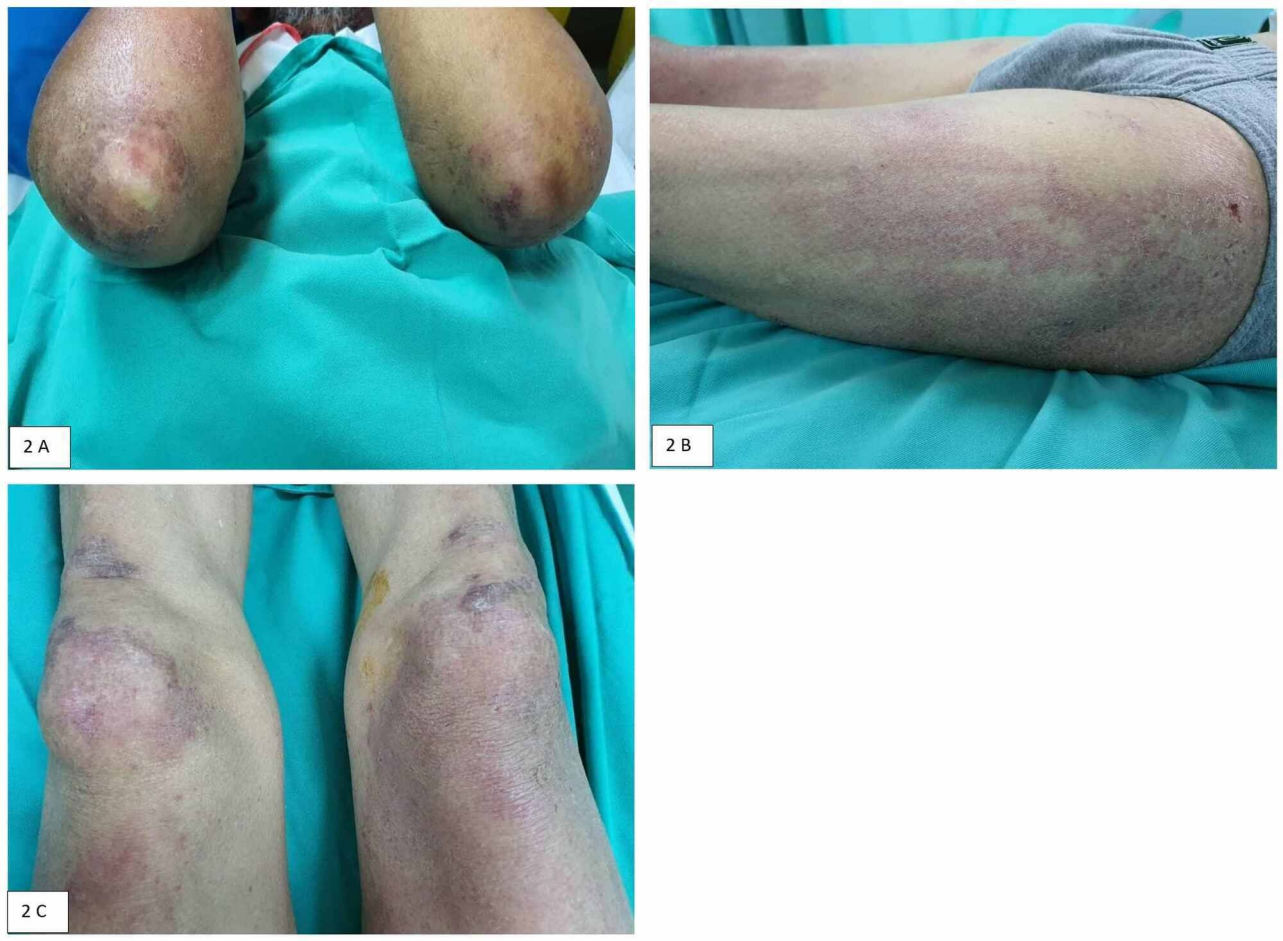
Photosensitive poikilodermatous eruptions on the extensors

Examination revealed heliotrope rashes over both of his eyebrows, Gottron’s papules at the knuckles of hands (Figure 1A, 1B), periungual telangiectasia (Figure 1B), and erythematous raised rashes over forearm extensors and lateral thighs (Figure 2A, 2B, 2C). His serum creatine kinase (CK) and lactate dehydrogenase (LDH) were elevated to 2859 U/L and 465 U/L, respectively. He was diagnosed with dermatomyositis based on his clinical and laboratory presentations. He was treated with prednisolone after skin biopsies and muscle biopsies were performed. His muscular and cutaneous symptoms responded well to steroid therapy.

**Figure 3:**
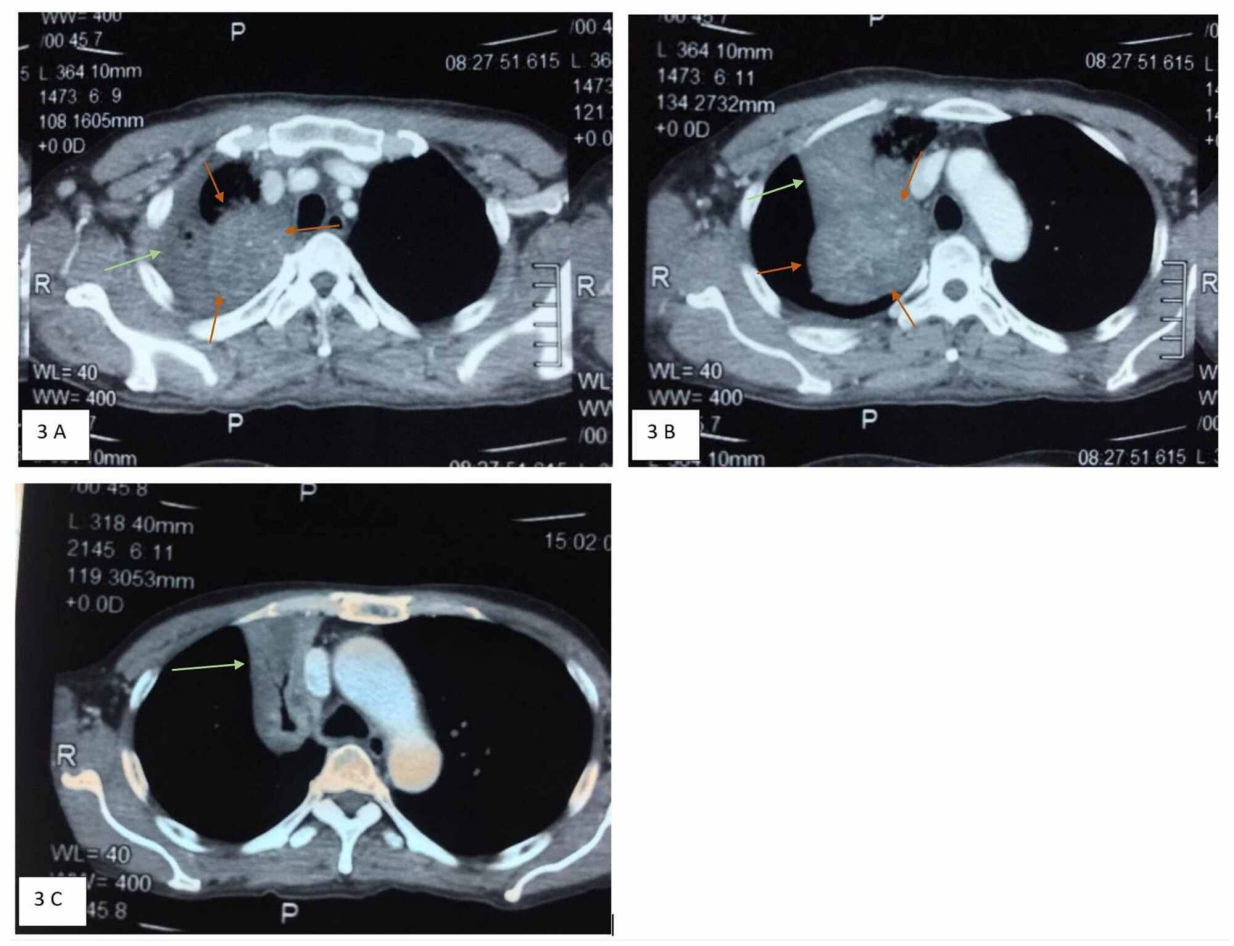
A, B) Ill-defined heterogeneous enhancing mass measured 6.5 cm x 7.1 cm in right upper lobe (red arrows). The mass obliterated the segmental branch of right upper lobe bronchus causing partial collapse of the right upper lobe (green arrows). C) Collapsed apical segment of right upper lobe (green arrow) with air bronchogram seen within. Collapsed part appeared smaller than in previous scan.

Chest radiograph on admission revealed right upper lobe collapse. Computed tomography (CT) thorax also showed similar findings with lung mass at the right upper lobe measuring 6.5 cm × 7.1 cm × 5.3 cm (Figure 3A, 3B, 3C). 

**Figure 4:**
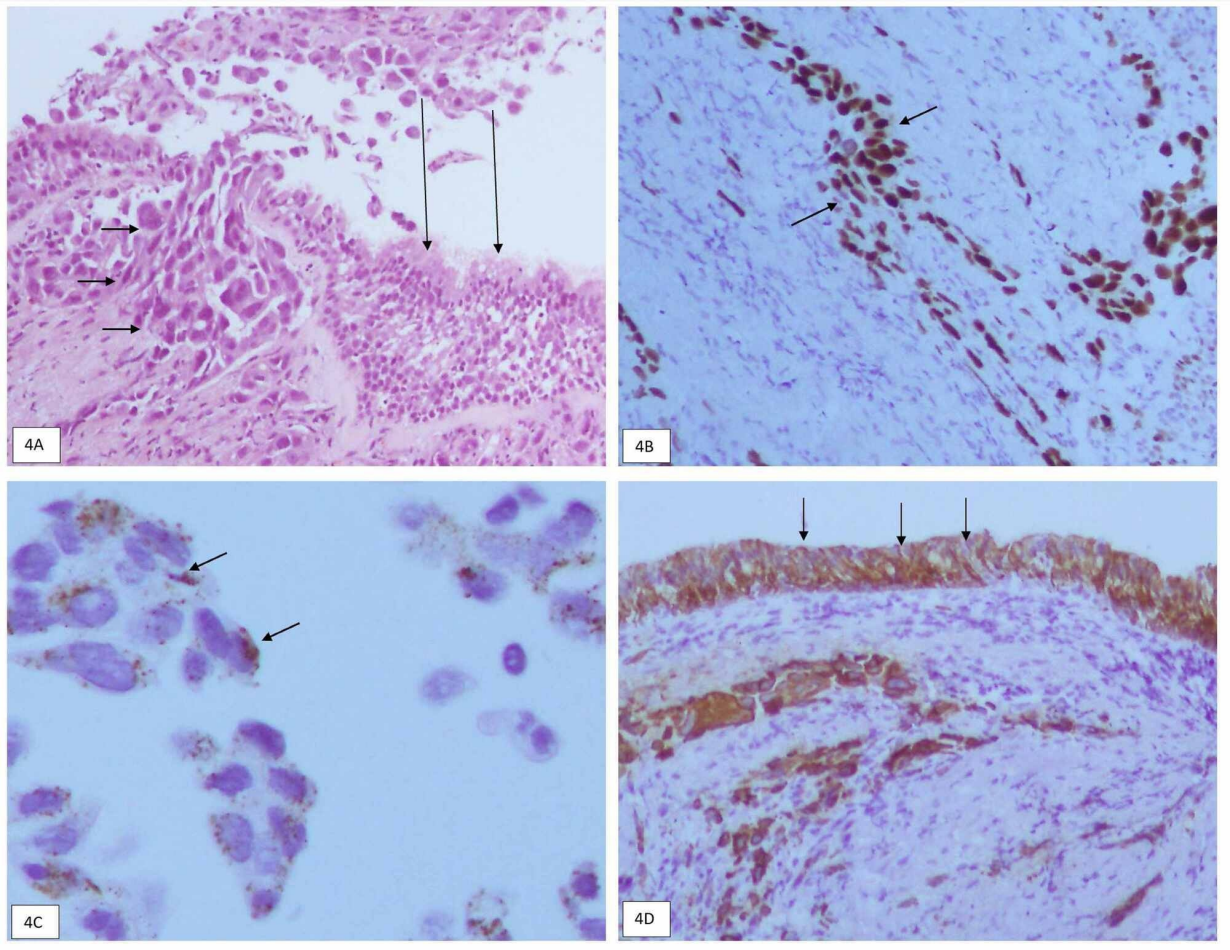
A) Histological examination (at x400). Normal respiratory epithelia (long arrows). Infiltration of malignant cells arranged in small nest and sheets displaying enlarged hyperchromatic pleomorphic nuclei with prominent nucleoli (short arrows). B) Immuno-histochemical stain for thyroid transcription factor 1 (TTF-1). C) Immuno-histochemical stain for Napsin A. D) Immuno-histochemical stain for creatine kinase (CK) 7.

Serial chest radiographs after a course of antibiotics showed expansion of collapsed right upper lobe with evidence of speculated mass at right middle zone. Bronchoscopy showed nodular lesions at right bronchiole of the right upper lobe. Biopsy of the nodules revealed the presence of lung malignant cells (Figure 4A). Also, immunohistochemical studies showed malignant cells that stained positive for thyroid transcription factor 1 (TTF-1) (Figure 4B), Napsin A (Figure 4C), and CK 7 (Figure 4D). These stained cells were negative for CK 20 and P40, in keeping with adenocarcinoma. 

## Discussion

Dermatomyositis is an idiopathic autoimmune disease characterized by skin and muscle lesions, with an incidence rate and female to male ratio of 0.6-1.0 per 100,000 individuals and 2:1, respectively [5,6]. 

There are many classification systems for dermatomyositis, of which the earliest was described by Peter and Bohan [7,8]. The diagnosis of dermatomyositis is considered definite, highly probable and possible when skin rash is associated with three, two, or one muscular criteria, which are as follows: (1) symmetric proximal muscle weakness, (2) elevation of serum skeletal muscle enzymes, (3) the electromyographic triad of typical finding, (4) muscle biopsy abnormalities (degeneration, regeneration, necrosis, phagocytosis and an interstitial mononuclear infiltrate), and (5) typical skin rash of dermatomyositis, including a heliotrope rash and Gottron’s sign/papules [8].

In our study, the patient was diagnosed with dermatomyositis as he fulfilled three of the above-mentioned criteria, i.e., symmetric proximal muscle weakness, elevation of serum skeletal muscle enzymes, and typical skin rash of dermatomyositis.

The onset of dermatomyositis has been frequently observed shortly before or after the detection of a malignant disease [9-11]. Our patient was diagnosed to have lung cancer after one year of dermatomyositis manifestation. A study that included 239 patients showed manifestation of malignancy within one year before or after diagnosis [12]. This suggests that some cases of dermatomyositis/polymyositis stem from an immune reaction to antigens, expressed in both cancer cells and muscles [5,6,10,13]. However, there are case series that reported a long interval between the onset of dermatomyositis and the diagnosis of lung cancer, ranging up to five years [14]. The pathogenesis of dermatomyositis in malignancy is unclear, but it has been hypothesized that the manifestation of dermatomyositis is an immunologic response to tumor-associated antigens.

Dermatomyositis is more commonly associated with small cell carcinoma than non-small cell carcinoma. Among the different histological types of lung cancer, dermatomyositis is found to have the highest incidence in small cell lung cancer, followed by squamous cell, and lastly adenocarcinoma [15].

Treatment of dermatomyositis is mainly by glucocorticoids, immunosuppressants, and removal of the underlying cause. Common immunosuppressants used in dermatomyositis are methotrexate, azathioprine, and mycophenolate mofetil. The main aim of treatment for dermatomyositis is to relieve the symptoms and prevent worsening of the disease. Several case studies demonstrated that complete resolution of dermatomyositis without steroid treatment after the underlying malignancy is treated. As for this patient, he was given oral prednisolone 0.5 mg/kg/day, and his musculocutaneous symptoms were markedly controlled. However, he will need further treatment pertaining to his lung adenocarcinoma, namely chemotherapy and possibly tyrosine kinase inhibitor if driver mutations are present.

## Conclusions

Cutaneous and musculoskeletal manifestations of dermatomyositis with the presence of respiratory symptoms can be the antecedent findings for the diagnosis of lung cancer. Hence, it warrants careful compilation of patient history, further investigations, and follow-ups.
